# Test–Retest Reliability of Running Economy and Other Physiological Parameters During 90 min of Running in Well‐Trained Male Endurance Runners

**DOI:** 10.1111/sms.70080

**Published:** 2025-05-27

**Authors:** Michele Zanini, Jonathan P. Folland, Richard C. Blagrove

**Affiliations:** ^1^ School of Sport, Exercise, and Health Sciences Loughborough University Loughborough UK; ^2^ School of Education, Childhood, Youth and Sport The Open University Milton Keynes UK; ^3^ National Institute for Health and Care Research (NIHR) Leicester Biomedical Research Centre Leicester UK

**Keywords:** durability, energy cost, fatigue, marathon, oxygen cost, physiological resilience

## Abstract

Running economy (RE) is highly reliable when measured in an unfatigued state; however, its reproducibility during prolonged exercise has not been investigated. RE is known to worsen during prolonged exercise (referred to as RE “durability”), and quantifying the reliability of RE durability measurements will determine our sensitivity to detect subtle differences between groups or conditions. This study aimed to assess the reliability of RE and other physiological parameters throughout a 90‐min run in the heavy‐intensity domain among well‐trained runners. Fourteen male marathon runners (maximal oxygen uptake (V̇O_2max_): 63.1 ± 5.8 mL·kg^−1^·min^−1^; marathon time: 02:47 ± 00:10 h:min) completed two 90 min treadmill runs at a speed of 10% Δ between lactate threshold one (LT) and two (LT2) (14.1 ± 0.9 km·h^−1^). Measurements were taken at 15 min intervals for RE (expressed as energy cost [EC] and oxygen cost [OC]), ventilation (V̇E), heart rate (HR), blood lactate (BLa), and rating of perceived exertion (RPE). Reliability was quantified using intraclass correlation coefficients (ICCs), coefficient of variation (CV), typical error (TE), and systematic differences. Both EC and OC demonstrated excellent reliability (ICCs 0.96–0.99; TEs 0.7%–1.4%; CVs 0.6%–1.2%) consistent across all time points (*p* > 0.15). V̇E and HR were highly reliable, whereas RPE and BLa exhibited greater variability, such that BLa became less reliable with run duration, especially after 60 min of exercise (time effect on BLa CV: *p* = 0.01). These results indicate that relatively modest changes in RE durability due to interventions or between conditions are likely to be detected, and provide critical information for future experiments aiming to measure and enhance the durability of endurance athletes.

## 1 Introduction

The classical model of endurance running suggests that performance can be predicted based on three physiological parameters: maximal oxygen uptake (V̇O_2max_), running economy (RE), and the fraction of sustainable V̇O_2max_ for a given distance [1]. Among these parameters, RE can be defined as the physiological cost of covering a given distance and is usually expressed as energy cost (EC, kcal·kg^−1^·km^−1^) or oxygen cost (OC, mL·kg^−1^·km^−1^). Although RE measurements have typically been made in the fresh state, logically RE throughout the duration of a prolonged run (e.g., of distance race duration) has a direct impact on performance because more economical runners can run at faster speeds for the same EC or OC [2]. Given the importance of RE throughout long‐distance races for performance modeling, it is crucial to establish the reliability of RE measurements during prolonged running.

Recently, the capacity of physiological determinants to avoid pronounced drifts during prolonged exercise has been named “durability” or “physiological resilience” [3, 4] and has been suggested as an independent factor determining endurance performance [3]. Reliability assessment of key performance determinants during prolonged exercise is therefore warranted to inform meaningful differences between athletes, conditions, and interventions. In this context, the durability of RE is of interest due to the widely documented upward drift in RE as fatigue accumulates [5, 6, 7, 8, 9], and the effect that this may have on endurance races such as the marathon [5]. Furthermore, the durability of RE appears to vary between runners of different levels [10], but the veracity of any inter‐individual differences may depend on the reliability of RE measurement throughout a bout of prolonged running.

In an unfatigued or fresh state, defined as a state with no running occurring in the several hours preceding an assessment, the reliability of RE has been assessed for both OC and EC in well‐trained runners, with typical error of measurement (TE) of 2.4%–4.7% for OC and 3.0%–3.8% for EC [11, 12, 13, 14, 15]. Furthermore, no differences have been found between the reliability of EC and OC measures of RE, with both having high intraclass correlation coefficients (ICCs, 0.74–0.83 [14]). However, no research has quantified the reliability of RE during prolonged running, and assessing RE reliability at different time points during prolonged exercise could therefore clarify whether the small day‐to‐day differences observed in fresh‐state tests [11, 14] persist, or are exacerbated, as fatigue develops. Furthermore, it is well‐known that prolonged exercise induces a shift in substrate utilization from carbohydrates (CHO) to fats [16] which may lead to different changes between EC and OC and could contribute to reliability discrepancies between these two measures, with a possible impact on RE durability. A high reliability of both EC and OC during prolonged running would support the use of either measure when reporting RE durability and confirm the small differences found in a recent study on well‐trained runners [10].

In addition to RE, physiological responses such as ventilation (V̇E), blood lactate (BLa), heart rate (HR), and rating of perceived exertion (RPE) provide valuable insights during prolonged exercise, and their reliability in a fresh state has been extensively assessed. For instance, BLa has a TE of 10%–20% [14, 17, 18], and HR has been shown to fluctuate between days by 2–8 beats·min^−1^ at the same sub‐maximal running speed [19, 20]. V̇E has also shown good to excellent ICCs during an unfatigued incremental test (0.89–0.95) while RPE has displayed lower reliability (0.37–0.88) [11, 17, 21]. Despite the above evidence, the test–retest reliability of these physiological parameters during a prolonged run is currently unknown.

Given the above background, this study aimed to assess the test–retest reliability of RE during two 90 min runs in well‐trained endurance runners, to examine time‐dependent changes in reliability throughout the trial, and to assess reliability differences between EC and OC measures of RE. Additionally, the reliability of other physiological parameters was assessed to provide a comprehensive understanding of their reproducibility during prolonged exercise. It was hypothesized that RE and other physiological parameters would be highly reliable throughout the prolonged run, although reliability would decrease as running duration progressed.

## 2 Methods

### 2.1 Participants

Seventeen male endurance runners, classified as Tier 2 and Tier 3 performance status [22], volunteered and gave written informed consent to participate in this study, which was approved by the Loughborough University Ethics Sub‐Committee. To be eligible to take part, participants had to be (i) aged 18–40 years, (ii) have run a marathon in < 3 h in the previous 6 months, (iii) be currently running ≥ 40 km·week^−1^, (iv) completing runs of ≥ 105 min in duration ≥ 2 times·month^−1^, and (iv) free of musculoskeletal injury. Prior to the first laboratory visit, participants completed a 4‐week training log and a training habit questionnaire, and reported their recent race performances (previous 6 months), which were verified via online databases.

### 2.2 Experimental Overview

Participants visited the laboratory on three occasions, with visits 1 and 2 separated by 5 ± 1 days (range 3–7), and visits 2 and 3 by 8 ± 2 days (range 7–14). Participants were asked to refrain from any caffeine and alcohol ingestion, and intense exercise, in the 24 h preceding each trial. All testing took place in the same physiology laboratory on a motorized treadmill (3DI, Treadmetrix, Utah, US), and participants completed the three visits at the same time of day (±1 h). The laboratory conditions were noted before each trial using a portable weather station (WS6730, Technoline, Germany) and were similar for all visits (temperature 19.8°C ± 1.1°C (range 18–21), relative humidity 47% ± 5% (range 45–55)). The first visit consisted of a discontinuous incremental treadmill running assessment to evaluate lactate threshold one (LT) and two (LT2), followed by a continuous incremental test to quantify V̇O_2max_. In the second and third visits, participants were required to perform two identical prolonged runs of 90 min both at the speed corresponding to 10% Δ between LT and LT2. Participants were required to replicate their diet and exercise routine in the 48 h before each visit and to wear the same footwear for all trials. Advanced footwear technology was not permitted, due to its ergogenic effect on RE [23]. The data presented in this manuscript were collected as part of a larger project on the effect of prolonged running on the durability of physiological determinants of endurance performance.

### 2.3 Visit 1: V̇O_2max_ and Lactate Threshold

Participants were given a diet record instructing them to report the time, food description, and weight of anything they consumed in the 48 h prior to visit 1, and were asked to replicate the same diet prior to the second and third trials for standardization. On the day of the trial, the athletes were instructed to replicate their typical high‐carbohydrate pre‐race dietary routine for further guidance. Upon arrival in the laboratory, participants' height and body mass (BM) were measured to the nearest 0.01 m and 0.01 kg on a stadiometer (Harpenden Stadiometer, Holtain Ltd., UK) and weight scale (Seca 700; Seca Hamburg, Germany), respectively. Participants then completed a 5 min warm‐up at a self‐selected speed before performing an incremental test on a treadmill consisting of 7–10 stages of 3 min, at speed increments of 1 km·h^−1^ per stage, until volitional exhaustion was reached. Each stage was interspersed with a 30 s recovery to allow the collection of a capillary blood sample from the earlobe for determination of BLa, with the speed of the first stage determined using participants' best race times and rounded to the nearest 1 km·h^−1^ (10.9 ± 0.9 km·h^−1^). The treadmill incline was kept at 1% to account for differences in air resistance compared to overground running [24]. RPE using the Borg 6 to 20 scale was measured, and HR was continuously recorded via a chest strap (H10, Polar, Kempele, FIN). LT, defined as the first rise in BLa from baseline, was calculated using a log–log analysis [25], whereas LT2 was calculated via the modified log–log Dmax method [26]. After the incremental test, participants passively rested for 5 min before commencing the continuous maximal test. The speed was set at 2 km·h^−1^ slower than the final speed reached on the discontinuous test, to facilitate a test duration between 5 and 8 min, with the test commencing at a gradient of 1%. Every minute thereafter the incline increased by 1% until volitional exhaustion was reached despite strong verbal encouragement to continue. Throughout both tests, participants wore a low‐dead‐space mask and breathed through an impeller turbine assembly (Jaeger Triple V, Jaeger GmbH, Hoechberg, Germany) to measure gas composition in inspired and expired air via an open‐circuit metabolic cart (Jaeger Vyntus CPX, Carefusion, San Diego, CA). The inspired and expired gas volume and concentration signals were continuously sampled, the latter using paramagnetic (O_2_) and infrared (CO_2_) analyzers (Jaeger Vyntus CPX, Carefusion, San Diego, CA) via a capillary line. These analyzers were calibrated before each test using a known gas mixture (16% O_2_ and 5% CO_2_) and ambient air. The turbine volume transducer was calibrated using a 3‐L syringe (Hans Rudolph, KS). The volume and concentration signals were time aligned, accounting for the transit delay in capillary gas and analyzer rise time relative to the volume signal. The metabolic cart was serviced and calibrated by the manufacturing company prior to the study.

### 2.4 Visit 2 and 3: 90 min Running Trial

Upon arrival, the participant's BM was recorded, and hydration status was measured via urine osmolality assessment (ATAGO PAL‐mOsm; Japan). Participants were then asked to stand quietly on the treadmill for 5 min to record respiratory gases at rest, and subsequently performed a 5 min warm‐up at a speed corresponding to 85% of LT. Thereafter, the speed was set to 10% Δ between LT and LT2, and participants ran for 90 min. Participants wore a mask throughout the test, and respiratory gases were sampled discontinuously for 5 min every 15 min utilizing the same method and equipment described for Visit 1. Participants straddled the treadmill belt for a few seconds before and after each sampling period to attach and detach the turbine assembly to the mask. HR (average of the last 30 s), BLa, and RPE were also sampled at 15 min intervals, and a 30 mL CHO solution (25% concentration of maltodextrin, to provide 30 g·h^−1^ of CHO) was given at the end of each measurement, at the end of which participants could drink water ad libitum. Fluid intake at the end of every 15 min interval was measured. Two motorized fans were positioned approximately 2 m behind and in front of the participant to provide a cooling effect throughout the trial and reduce the risk of hyperthermia.

### 2.5 Measurements

#### 2.5.1 Blood Lactate

Capillary blood was collected via a 20‐μL capillary tube from the participant's ear lobe. The sample was immediately haemolysed in a solution tube and assessed for BLa concentration (Biosen C‐Line, EKF Diagnostics, Cardiff, UK).

#### 2.5.2 Maximal Measures

Breath‐by‐breath V̇O_2_ data were continuously recorded and initially filtered to exclude errant breaths, defined as values lying more than 4 standard deviations (SD) from the local mean (previous 5 breaths), and subsequently converted to second‐by‐second data using linear interpolation. V̇O_2max_ was defined as the highest 30 s moving average from the V̇O_2_ data. The presence of a V̇O_2_ plateau was also visually inspected and was considered to be attained if V̇O_2_ remained unchanged for > 30 s during the ramp incremental test. All participants reached a V̇O_2_ plateau during the test.

#### 2.5.3 Running Economy

During the prolonged runs, the average of V̇O_2_ and V̇CO_2_ data was collected in the final 2 min of each 15 min stage and used to calculate OC and EC. OC was expressed as mL·kg^−1^·km^−1^ and EC was calculated based on the method described by Shaw et al. [14]. Briefly, updated nonprotein respiratory quotient equations [27] were used to estimate substrate utilization (g·min^−1^). The energy derived from each substrate was calculated by multiplying fat and carbohydrate utilization by 9.75 and 4.07 kcal, respectively [28]. Absolute EC was calculated as the sum of the energy derived from fat and carbohydrate expressed as kcal·kg^−1^·km^−1^. To account for changes in BM during the prolonged runs, BM pre‐ to post‐trial was measured and linear regression was used to estimate BM at each measurement time point. The adjusted BM was used to calculate RE at each time point.

### 2.6 Statistical Analysis

All data are presented as mean ± SD. The Greenhouse–Geisser correction was applied when the assumption of sphericity was violated. Normal distributions of the dependent variables were confirmed via Shapiro–Wilk tests. A two‐way repeated‐measure analysis of variance (ANOVA; trial × time and main effect of trial) was used to compare differences in physiological response between the two 90 min runs for EC, OC, BLa, HR, RPE, VE, respiratory exchange ratio (RER), BM, and fluid intake. Post hoc analysis with Bonferroni adjustment was used to identify the origin of any significant difference. Differences in BM and urine osmolality at the start of the test were analyzed via a paired *t*‐test.

To assess intra‐individual variation between tests, the TE, a value that encompasses both technical and biological variation, was calculated using the root mean squares error method. Within‐subject variation between trials was also calculated for each measure and collection time during the 90‐min trial, as coefficient of variation (CV) for each individual ([Standard deviation/mean]*100). A one‐way repeated‐measure ANOVA was used to assess changes in reliability for each physiological measure during the 90‐min trial by comparing the CVs across time points. ICCs (two‐way random, single measure) were also calculated for each measure as an indicator of reliability [29] including a 95% confidence interval (CI). ICCs are interpreted along a spectrum from poor (> 0.5) to moderate (0.5–0.75), good (0.75–0.9), and excellent (> 0.9) according to their associated 95% CIs (e.g., a 95% CI of 0.6–0.8 would be interpreted as moderate to good reliability). The threshold for significance was fixed at *p* < 0.05. All statistical analyses were performed using SPSS version 28 (SPSS Inc., Chicago, IL, USA).

## 3 Results

Of the 17 participants who began at least one prolonged run, 14 completed the study. One participant could not complete the runs due to premature exhaustion, and 2 runners only completed one run due to calf muscle discomfort. A summary of participant characteristics at baseline is shown in Table 1. Participants completed the prolonged runs at 14.1 ± 0.9 km·h^−1^, at an initial intensity corresponding to 79.3% ± 4.5% V̇O_2max_, covering 21.2 ± 1.4 (19.5–24.5 km) over the 90‐min trial. Pre‐run BM did not differ between trials (68.1 ± 7.5 vs. 68.5 ± 7.1 kg; *p* = 0.20; TE 0.7%), and changes were the same following the 90‐min run (−2.1 ± 0.7 vs. −2.0% ± 0.5%; *p* = 0.71). Urine osmolality before the runs was also the same between trials (397 ± 313 vs. 416 ± 307 mOsm·kg^−1^; *p* = 0.73).

**TABLE 1:** Participant characteristics (*n* = 14).

Age (year)	Height (m)	Body mass (kg)	Marathon time (h:min)	T. volume (km·w^−1^)	V̇O_2max_ (mL·kg^−1^·min^−1^)	LT speed (km·h^−1^)	LT2 speed (km·h^−1^)	Trial speed (km·h^−1^)
35. ± 8.6	1.76 ± 0.08	68.7 ± 7.8	2:46 ± 0:10	79 ± 23	63.1 ± 5.8	14.0 ± 0.9	16.7 ± 1.1	14.1 ± 0.9

*Note:* Marathon time: best time in the 6 months before visit 1; T. volume: training volume; V̇O_2max_: maximal oxygen uptake. Data are expressed as mean ± SD.

**FIGURE 1:**
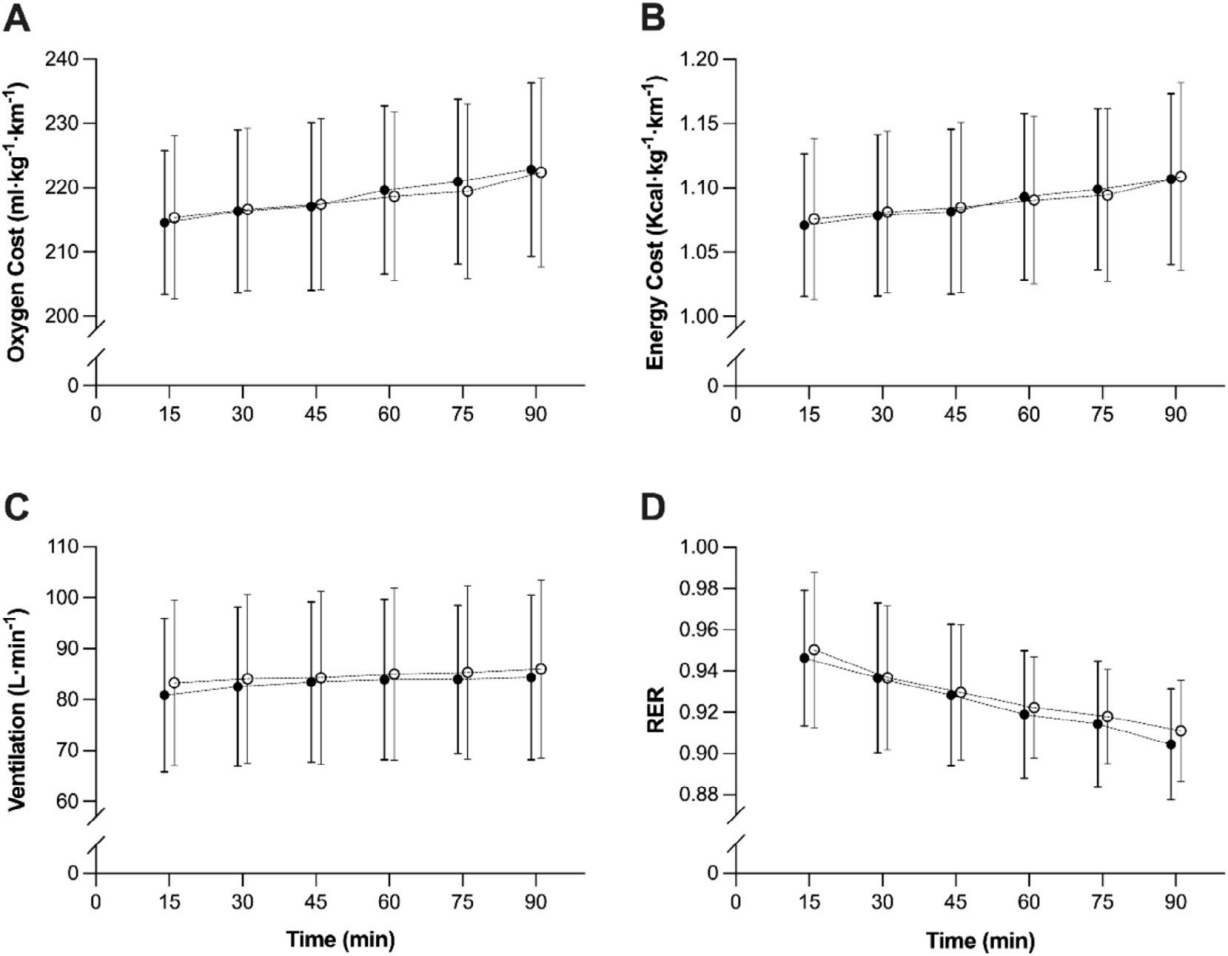
Oxygen cost (A), energy cost (B), ventilation (C) and RER (D) during the two 90 min runs. Filled circles indicate the first run, and open circles indicate the second run. Data are mean ± SD (*n* = 14).

**FIGURE 2:**
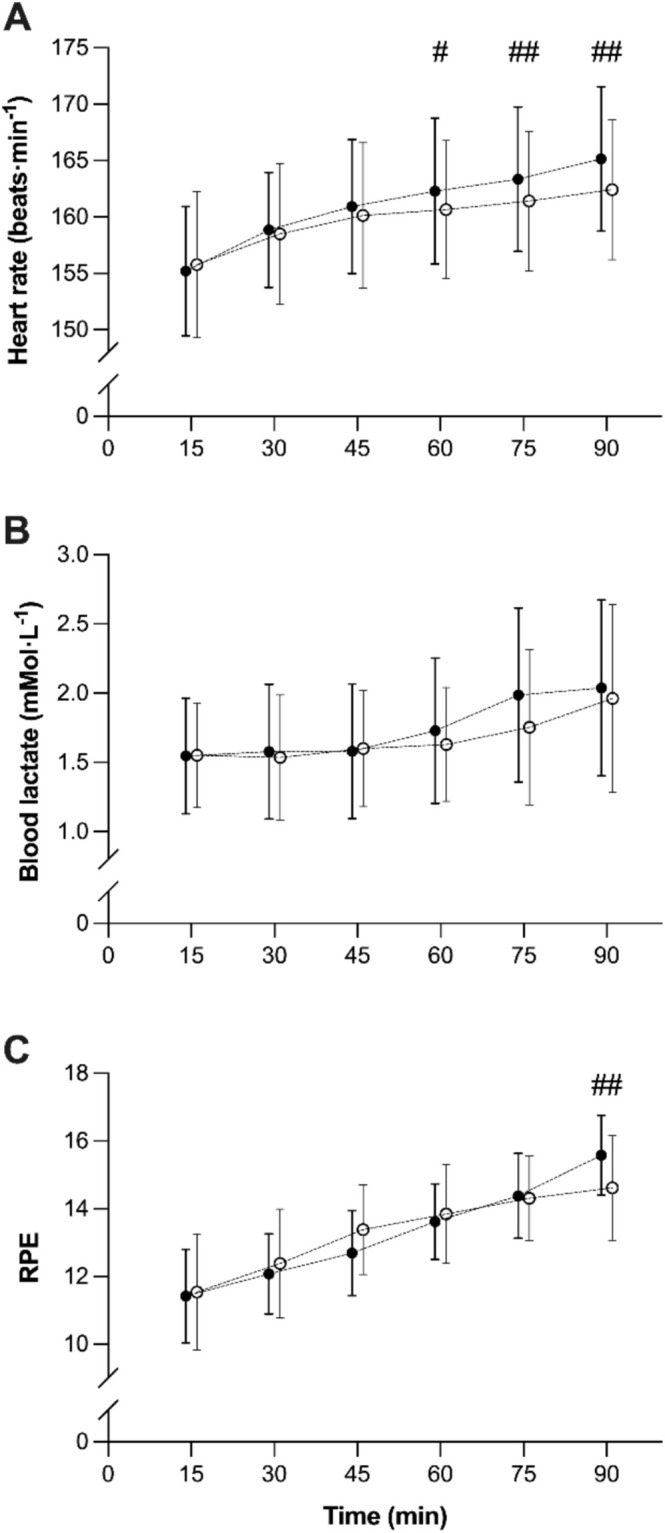
Heart rate (A), Blood lactate (B), and RPE (C) during the two 90 min runs. Filled circles indicate the first trial, and open circles indicate the second. Differences between trials at a specific time point are indicated by: # *p* < 0.05, ## *p* < 0.01. Data are mean ± SD (*n* = 14).

From ANOVA analysis, no systematic differences were found for EC or OC during the 90‐min run, with no main effect of trial (*p* = 0.79 (EC), *p* = 0.78 (OC); Figure 1) nor trial × time interaction effect (*p* = 0.34 (EC), *p* = 0.17 (OC); Figure 1). For fluid intake, there was no main effect of trial (*p* = 0.73) or trial × time interaction (*p* = 0.86), indicating that BM adjustments for EC and OC were not confounded by differences in fluid intake between trials. Similarly, there were no systematic differences between the physiological responses to the two 90 min runs for V̇E, RER, and Bla (*p* = 0.12–0.82; Figures 1 and 2). No main effect of trial was found for HR (*p* = 0.06); however, a trial × time interaction occurred (*F* = 5.20, *p* = 0.01, *η*_p_^2^ = 0.29) with lower HR in the second trial at 60 (*p* = 0.04), 75 and 90 min, compared to 15 min (*p* = 0.01; Table 4; Figure 2). Similarly, there was no main effect of trial for RPE (*p* = 0.84), but a trial × time interaction effect (*F* = 3.54, *p* = 0.01, *η*_p_^2^ = 0.23), with a lower RPE in trial 2 at 90 min (*p* = 0.01; Table 4; Figure 2). Individual responses to the two 90 min trials for OC, EC, BLa, and HR are represented in Figure S1.

Within‐participant variation between the two runs was very low for both EC and OC (CV 0.6%–1.2%; TE ≤ 1.4%; Table 2), and with no effect of time on the CV of either measure during the 90‐min run (EC: *F* = 1.47, *p* = 0.21, *η*_p_^2^ = 0.10; and OC: *F* = 1.95, *p* = 0.15, *η*_p_^2^ = 0.13). Similarly, low within‐participant variability was found for V̇E (TE ≤ 4.0%), RER (TE ≤ 1.9%), and HR (TE ≤ 1.3%), with CVs ranging from 0.9% to 3.6% (Tables 2 and 3) and no time effect for CV during the run (*p* > 0.34, *η*_p_^2^ < 0.08) except for HR, showing an increased CV as the trial progressed (*p* = 0.02, *η*_p_^2^ = 0.19). RPE displayed moderate variability between trials (TE 5.4%–8.6%) with a CV of 4.2%–6.0%, which remained stable throughout the run (*p* = 0.78, *η*_p_^2^ = 0.02). BLa also showed moderate variation up to 60 min (TE 7%–14%; CV 5%–9%) and increased further at 75 and 90 min (TE 20%–22%; CV 14%–16%; Table 4), with a time effect of CV during the course of the run (*p* = 0.01, *η*_p_^2^ = 0.20). The effect of time on CVs for HR and BLa indicates that the reliability of these measures decreased during the prolonged run, although post hoc analysis could not detect differences between specific time points.

**TABLE 2:** Reliability of running economy measurements collected during the 90 min runs.

Measure	Time (min)	Trial 1	Trial 2	CV (%)	TE	TE (%)	ICC (95% CI)
Energy cost (Kcal·kg^−1^·km^−1^)	15	1.07 ± 0.06	1.08 ± 0.06	0.7	0.01	0.7	0.98 (0.94–0.99)***
	30	1.08 ± 0.06	1.08 ± 0.06	0.6	0.01	0.7	0.99 (0.97–1.00)***
	45	1.08 ± 0.06	1.08 ± 0.07	1.0	0.01	1.3	0.96 (0.89–0.99)***
	60	1.09 ± 0.06	1.09 ± 0.07	0.9	0.01	1.1	0.97 (0.90–0.99)***
	75	1.10 ± 0.06	1.09 ± 0.07	0.9	0.01	1.2	0.96 (0.88–0.99)***
	90	1.11 ± 0.07	1.11 ± 0.07	0.7	0.01	1.0	0.98 (0.93–0.99)***
	Mean	1.09 ± 0.06	1.09 ± 0.07	0.8	0.01	1.0	0.97 (0.96–0.98)***
Oxygen cost (mL·kg^−1^·km^−1^)	15	215 ± 11	215 ± 13	0.7	2	0.8	0.98 (0.94–0.99)***
	30	216 ± 13	217 ± 13	0.6	2	0.7	0.99 (0.98–1.00)***
	45	217 ± 13	217 ± 13	1.0	3	1.3	0.96 (0.87–0.99)***
	60	220 ± 13	219 ± 13	1.0	3	1.2	0.96 (0.89–0.99)***
	75	221 ± 13	219 ± 14	1.2	3	1.4	0.96 (0.88–0.99)***
	90	223 ± 13	222 ± 15	0.9	3	1.2	0.96 (0.89–0.99)***
	Mean	219 ± 13	218 ± 13	0.9	3	1.1	0.96 (0.95–0.98)***

*Note:* Data are displayed as mean ± SD (*n* = 14). ICC significance: ***p < 0.001.

Abbreviations: CI, confidence interval; CV, within‐participant coefficient of variation; ICC, intraclass correlation coefficient; TE, typical error.

**TABLE 3:** Reliability of ventilatory measurements collected during the 90 min runs.

Measure	Time (min)	Trial 1	Trial 2	CV (%)	TE	TE (%)	ICC (95% CI)
Ventilation (L·min^−1^)	15	80.9 ± 15.1	83.3 ± 16.2	3.6	3.1	3.7	0.97 (0.91–0.99)***
	30	82.6 ± 15.6	84.1 ± 16.5	2.8	2.6	3.1	0.97 (0.91–0.99)***
	45	83.4 ± 15.7	84.3 ± 17.0	3.1	3.0	3.5	0.97 (0.90–0.99)***
	60	84.0 ± 15.7	85.0 ± 16.9	3.0	3.0	3.5	0.97 (0.90–0.99)***
	75	84.0 ± 14.6	85.3 ± 17.0	3.5	3.4	4.0	0.97 (0.90–0.99)***
	90	84.3 ± 16.2	86.0 ± 17.5	2.8	3.0	3.5	0.97 (0.90–0.99)***
	Mean	83.2 ± 15.1	84.7 ± 16.4	3.1	2.9	3.5	0.96 (0.94–0.98)***
Respiratory exchange ratio (AU)	15	0.95 ± 0.03	0.95 ± 0.04	1.0	0.01	1.5	0.85 (0.60–0.95)***
	30	0.94 ± 0.04	0.94 ± 0.03	1.2	0.01	1.5	0.85 (0.59–0.95)***
	45	0.93 ± 0.03	0.93 ± 0.03	1.4	0.02	1.6	0.80 (0.49–0.93)***
	60	0.92 ± 0.03	0.92 ± 0.02	1.5	0.02	1.9	0.63 (0.16–0.86)**
	75	0.91 ± 0.03	0.92 ± 0.02	1.5	0.02	1.9	0.59 (0.10–0.85)**
	90	0.90 ± 0.03	0.91 ± 0.02	1.5	0.02	1.8	0.60 (0.15–0.85)**
	Mean	0.92 ± 0.03	0.93 ± 0.03	1.3	0.02	1.7	0.78 (0.68–0.85)***

*Note:* Data are displayed as mean ± SD (*n* = 14). ICC significance: **p < 0.01, ***p < 0.001.

Abbreviations: AU, arbitrary unit; CI, confidence interval; CV, within‐participant coefficient of variation; ICC, intraclass correlation coefficient; TE, typical error.

**TABLE 4:** Reliability of heart rate, blood lactate, and RPE measurements collected during the 90 min runs.

Measure	Time (min)	Trial 1	Trial 2	CV (%)	TE	TE (%)	ICC (95% CI)
Heart rate (beats·min^−1^)	15	155 ± 6	156 ± 6	1.1	2	1.3	0.89 (0.70–0.96)***
	30	159 ± 5	159 ± 6	0.9	2	1.1	0.92 (0.76–0.97)***
	45	161 ± 6	160 ± 6	0.9	2	1.1	0.91 (0.76–0.97)***
	60	162 ± 6	161 ± 6^#^	1.1	2	1.2	0.88 (0.62–0.96)***
	75	163 ± 6	161 ± 6^##^	1.1	2	1.0	0.89 (0.53–0.97)***
	90	165 ± 6	162 ± 6^##^	1.6	2	1.3	0.82 (0.28–0.95)***
	Mean	161 ± 7	160 ± 6	1.1	2	1.3	0.89 (0.82–0.94)***
Blood lactate (mMol·L^−1^)	15	1.55 ± 0.42	1.55 ± 0.38	5.3	0.13	8.4	0.90 (0.72–0.97)***
	30	1.58 ± 0.49	1.54 ± 0.45	8.8	0.17	11.2	0.87 (0.64–0.96)***
	45	1.58 ± 0.49	1.60 ± 0.42	6.6	0.11	7.1	0.94 (0.83–0.98)***
	60	1.73 ± 0.52	1.63 ± 0.41	7.6	0.23	13.7	0.76 (0.42–0.91)***
	75	1.99 ± 0.63	1.75 ± 0.56	16.5	0.42	22.7	0.48 (−0.11–0.79)**
	90	2.04 ± 0.64	1.96 ± 0.68	14.2	0.39	19.6	0.66 (0.21–0.88)*
	Mean	1.74 ± 0.56	1.67 ± 0.50	9.8	0.27	15.9	0.73 (0.62–0.82)***
Rating of perceived exhaustion (6–20)	15	11.4 ± 1.4	11.5 ± 1.7	5.3	1.0	8.5	0.62 (0.11–0.87)*
	30	12.1 ± 1.2	12.4 ± 1.6	6.1	1.1	8.6	0.45 (−0.11–0.79)
	45	12.7 ± 1.3	13.4 ± 1.3	4.4	0.8	6.4	0.52 (0.03–0.82)*
	60	13.6 ± 1.1	13.8 ± 1.5	4.2	0.9	6.7	0.51 (−0.03–0.82)*
	75	14.4 ± 1.3	14.3 ± 1.3	4.8	1.0	6.8	0.41 (−0.19–0.78)
	90	15.6 ± 1.2	14.6 ± 1.6^##^	5.2	0.8	5.4	0.53 (−0.01–0.83)**
	Mean	13.3 ± 1.9	13.3 ± 1.8	5.0	1.0	7.3	0.72 (0.59–0.81)***

*Note:* Data are displayed as mean ± SD (*n* = 14). Different from Trial 1: #*p* < 0.05, ##*p* < 0.01. ICC significance: *p < 0.05, **p < 0.01, ***p < 0.001.

Abbreviations: CI, confidence interval; CV, within‐participant coefficient of variation; ICC, intraclass correlation coefficient; TE, typical error.

ICC analysis revealed significant and strong relationships between trials for all the physiological variables assessed at each trial time point except for RPE at 30 (*p* = 0.06) and 75 min (*p* = 0.08) when the two trial values were unrelated (Tables 2, 3, 4). No notable differences were found when expressing RE as EC or OC, with excellent ICCs of 0.96–0.99 for both measures that were consistent throughout both trials.

## 4 Discussion

This study investigated the test–retest reliability for RE and other physiological markers throughout a 90‐min run (i.e., time‐dependent effects) in the heavy intensity domain and specifically compared the reliability of EC and OC. EC and OC both showed similar and very high reliability throughout the prolonged run (CV ≤ 1.2%), with no differences between the within‐participant CVs of these measures at any time point. This indicates that both EC and OC can be used to accurately measure RE changes during prolonged running, which is of importance for studies investigating “durability” or “physiological resilience”. V̇E, RER, and HR were also highly reliable, whereas RPE and BLa showed low to moderate reliability through the run, particularly after 60 min.

This study is the first to demonstrate that RE is highly reliable throughout prolonged running, with similar test–retest reliability of EC and OC as measures of RE. EC and OC showed excellent ICCs (0.96–0.99), with small TEs (≤ 1.4%) and CVs (≤ 1.2%). There was no effect of time (i.e., measurement time point during the prolonged run) on the CV of EC and OC, demonstrating the consistent reliability of these measures throughout prolonged running. All the reliability metrics for EC and OC varied minimally throughout the run, by only 0.03 for ICC, 0.7% for TE, and 0.5% for CV. Although no other research has assessed the reliability of RE during prolonged running, one study investigating the OC response over 15, 32, and 42 km reported comparable changes across repeated trials [5], which is aligned with our findings. Given the recent interest in physiological changes during prolonged exercise (i.e., “durability” [3, 4]), the high reliability of EC and OC is particularly important. These results suggest that even small differences in RE durability—proposed to influence endurance performance—are likely detectable between diverse runners, conditions, and in response to interventions. This also has practical implications for applied performance settings, where reliable physiological measures are essential for assessing longitudinal responses to training.

The high reliability of both EC and OC was higher than previous reports of measurements in fresh/unfatigued conditions [14] and similar to or better than other studies that just measured OC [12, 15, 30]. For OC, the TE (0.7%–1.4%) found in this study is in line with previous results in highly trained runners (1.3%–1.8%) where diet and exercise were controlled [11, 15, 30]. Conversely, studies that did not control for diet or exercise have reported lower ICCs (0.73–0.84) and higher TEs (2.3%–3.8%) [13, 14]. The reliability of EC in a fresh state has only been investigated in two other studies in highly trained runners, reporting a TE of 1.2%–1.6%—similar to the current investigation (0.7%–1.3%)—when diet was controlled for [11] and of 3.1%–3.7% when no controls were employed [14]. The low TE found confirms findings from previous studies and indicates that OC and EC continue to be highly reliable over longer periods of running.

The high reliability found in this study could be partially due to the within‐participant standardization of diet and exercise in the 48 h preceding each trial, allowing similar energy availability during each run. The collection time for RE analysis was also longer than the sampling duration of most previous studies (2 vs. 1 min) and likely helped to minimize the impact of erroneously collected breaths. Furthermore, the longer time spent at a consistent speed, compared to the 3–8 min duration used to assess RE reliability in previous studies [13, 14], could also explain inter‐study differences. This longer duration may have ensured a stabilization of both V̇O_2_ and V̇CO_2_, which is important considering that V̇CO_2_ takes longer to reach a steady state [31] and may have an influence on the measure of EC, which depends on both V̇O_2_ and V̇CO_2_ [32]. It is also important to note that the reliability of respiratory measures such as V̇O_2_, V̇CO_2_, and V̇E is highly dependent on the type of metabolic cart used to collect data. The Jaeger Vyntus used in the current study is known to be highly valid and reliable [33]. The lack of a time effect for CVs throughout the 90‐min run implies that the upward drift in RE (expressed as either OC or EC) during prolonged exercise can be an accurate measure to assess changes in durability in response to interventions in well‐trained endurance runners. Importantly, RE is only one of the physiological determinants of endurance performance; therefore, assessing the reliability of V̇O_2max_ and physiological thresholds following prolonged exercise would also be of interest.

The reliability of the other physiological measures during prolonged running has not previously been investigated. V̇E was highly reliable (ICCs 0.96–0.97; TE < 4.0%; CV < 3.6%) and showed consistency throughout the trial. Our results are in line with previous investigations assessing V̇E during an unfatigued step test, where CVs ranged from 2% to 4% and ICCs > 0.89 [17, 21]. Another study found higher TE (6%–8%) in elite runners [13], possibly due to the lack of control over diet and training between trials. The high reproducibility found for HR (ICCs 0.82–0.92, TE ≤ 1.3%) is similar to previous reports during incremental sub‐maximal tests with trained runners [11, 13, 17, 34]. HR reliability was also higher than studies with recreational runners (ICC: 0.47–0.79, TE: 1.6%–4.3%) despite their standardization of diet and exercise [35, 36], which suggests that training status may influence the reliability of HR. It is important to note that a bias was found from 60 min onwards, with a significantly reduced HR (1–3 beats·min^−1^) in the second trial, indicating that athletes may have become more accustomed to the prolonged run on the treadmill. However, HR was only 1–3 beats·min^−1^ lower in the later stages of the second run, which is below the reported daily HR variation at standardized intensity in a fresh state [19]. This suggests that the inter‐trial difference in HR has likely little influence on the overall physiological response to the run.

BLa showed more moderate reliability up to 45 min (ICCs 0.87–0.94, TE ≤ 11%) but declined further from 60 min onwards (ICCs 0.48–0.76, TE 14%–23%) confirmed by the time effect of CVs, although no differences were found between any time points. The reliability of BLa measurements during incremental tests in previous studies controlling for diet and exercise was similar to our results, with a TE ranging from 6% to 24% [11, 17]. It is noteworthy that although the reliability of BLa was low, the absolute differences between trials were very small (< 0.2 mMol·L^−1^) and bias was absent independently of run time. In a practical context, this difference may be of little importance as only a larger increase in BLa (i.e., > 0.5 mMol·L^−1^) may suggest a change above the analyzer's measurement error and have meaningful physiological consequences in response to exercise. The reliability of RPE was moderate, with ICCs ranging from poor to good (0.41–0.72), a TE of 5.4%–8.6%, and consistently moderate CVs during the run (4.2%–6.1%). Previous studies assessing incremental tests have reported an intensity‐dependent reliability of RPE, with a tendency to improve as speed increases [11, 17, 34]. At RPEs similar to this study, the reliability was in line with our findings (ICCs 0.36–0.58; TE 4.9%; CV 6.5%) [11, 17]. RPE reliability has been suggested to improve over several trials [37], indicating that athletes may need longer exposure to the testing conditions to adjust their RPE compared to the other physiological variables, which do not rely on a subjective interpretation. During prolonged running, the RPE response to two time‐to‐exhaustion runs at 70% V̇O_2max_ showed a similar pattern to this investigation, with a slight RPE reduction at 90 min in the second trial, although reliability was not quantified [38].

This study is not without limitations. Firstly, the study only included male participants, which limits the ability to extrapolate these findings to female athletes, considering that differences in the physiological response to prolonged exercise have been suggested between sexes [39]. The decision to only include males was based on the currently unknown effect of the menstrual cycle phase (MCP) on RE during prolonged running, and the unclear effect of MCP on RE in an unfatigued state, with studies showing RE to be better in the mid‐luteal [40, 41], follicular phase [42], and unaffected by MCP [43], with a large inter‐individual variability in RE at different MCP. Although we recognize that MCP could have been standardized, this would have required testing sessions to be spaced by approximately 1 month to account for phase‐specific variations. Such a design would have likely introduced training‐induced adaptations over time, confounding the interpretation of our results. Future research investigating the effect of MCP on RE durability and its reliability would be fundamental to extending our findings to female runners. Furthermore, only well‐trained marathon runners were recruited, thus these results may not be generalizable to runners of a different performance level or to other populations not accustomed to prolonged running. The study was conducted under tightly controlled laboratory conditions, with between‐trial standardization of diet, exercise, and environmental factors. Although this level of control is useful for minimizing confounding variables and maximizing measurement accuracy in experimental trials, it may not fully reflect the variability present in more ecologically valid settings (e.g., marathon races) where these factors are less consistent. The test–retest design of this study offers important insights into the consistency of physiological measures during prolonged exercise. However, including a third trial could help verify or challenge the observed bias in HR and RPE towards the end of the prolonged run.

## 5 Perspective

This study assessed the test–retest reliability of RE and other physiological markers during a 90‐min run in the heavy intensity domain in well‐trained male endurance runners, revealing consistently high reliability for both EC and OC throughout the duration of the run. V̇E, RER, and HR also showed high reliability, though HR exhibited a minor reduction in the second trial from 60 min. BLa and RPE were less reliable, with BLa becoming particularly variable after 60 min and RPE showing moderate reliability throughout each trial. Overall, this study provides the first insight into the reliability of RE and other physiological measures during prolonged running. The high reliability of RE during the 90‐min run indicates that even relatively subtle differences in RE durability between runners or conditions, and alterations due to acute or chronic interventions, may be detectable. These results are of particular importance for current and future studies investigating durability in endurance athletes, particularly when assessing differences between conditions or longitudinally over time.

## Author Contributions

M.Z., R.C.B., and J.P.F. conceived the study; M.Z. collected the data; M.Z. analyzed the data and wrote the initial manuscript draft; M.Z., R.C.B., and J.P.F. contributed to the draft updates. All authors approved the final version.

## Conflicts of Interest

The authors declare no conflicts of interest.

## Supporting information


**Figure S1.** Individual responses for oxygen cost (A), energy cost (B), blood lactate (C) and heart rate (D) during the two 90 min runs. Open circles indicate the first run, and open triangles the second. Each participant is represented by different color (*n* = 14).

## Data Availability

Data supporting the results presented in the manuscript are included in the figures and online resources whenever possible, and are available upon request to the corresponding author.
