# Supplementary material for: Test–Retest Reliability of Running Economy and Other Physiological Parameters During 90 min of Running in Well‐Trained Male Endurance Runners
Source: Scand J Med Sci Sports. 2025 May 27;35(6):e70080. doi: 10.1111/sms.70080 (PMC12107507; doi:10.1111/sms.70080)
Supplement: Supplementary file 1 — Figure S1. Individual responses for oxygen cost (A), energy cost (B), blood lactate (C) and heart rate (D) during the two 90 min runs. Open circles indicate the first run, and open triangles the second. Each participant is represented by different color (n = 14). [file SMS-35-e70080-s001.docx]

**Supplementary Materials**

**Test-retest reliability of running economy and other physiological parameters during 90 minutes of running in well-trained male endurance runners**

**Jorunal:** Scandinavian Journal of Medicine and Science in Sports

**Authors:**

Michele Zanini^1,2^, Jonathan P. Folland^1,3^, Richard C. Blagrove^1^

1. School of Sport, Exercise, and Health Sciences; Loughborough University (UK)

2. School of Education, Childhood, Youth and Sport; The Open University (UK)

3. National Institute for Health and Care Research (NIHR); Leicester Biomedical Research Centre, Leicester (UK)

**Corresponding author:**

Michele Zanini (ORCID: 0009-0007-8148-8843)

School of Sport, Exercise & Health Sciences

Loughborough University, LE11 3TU, United Kingdom

E-mail: [m.zanini@lboro.ac.uk](mailto:m.zanini@lboro.ac.uk)

**
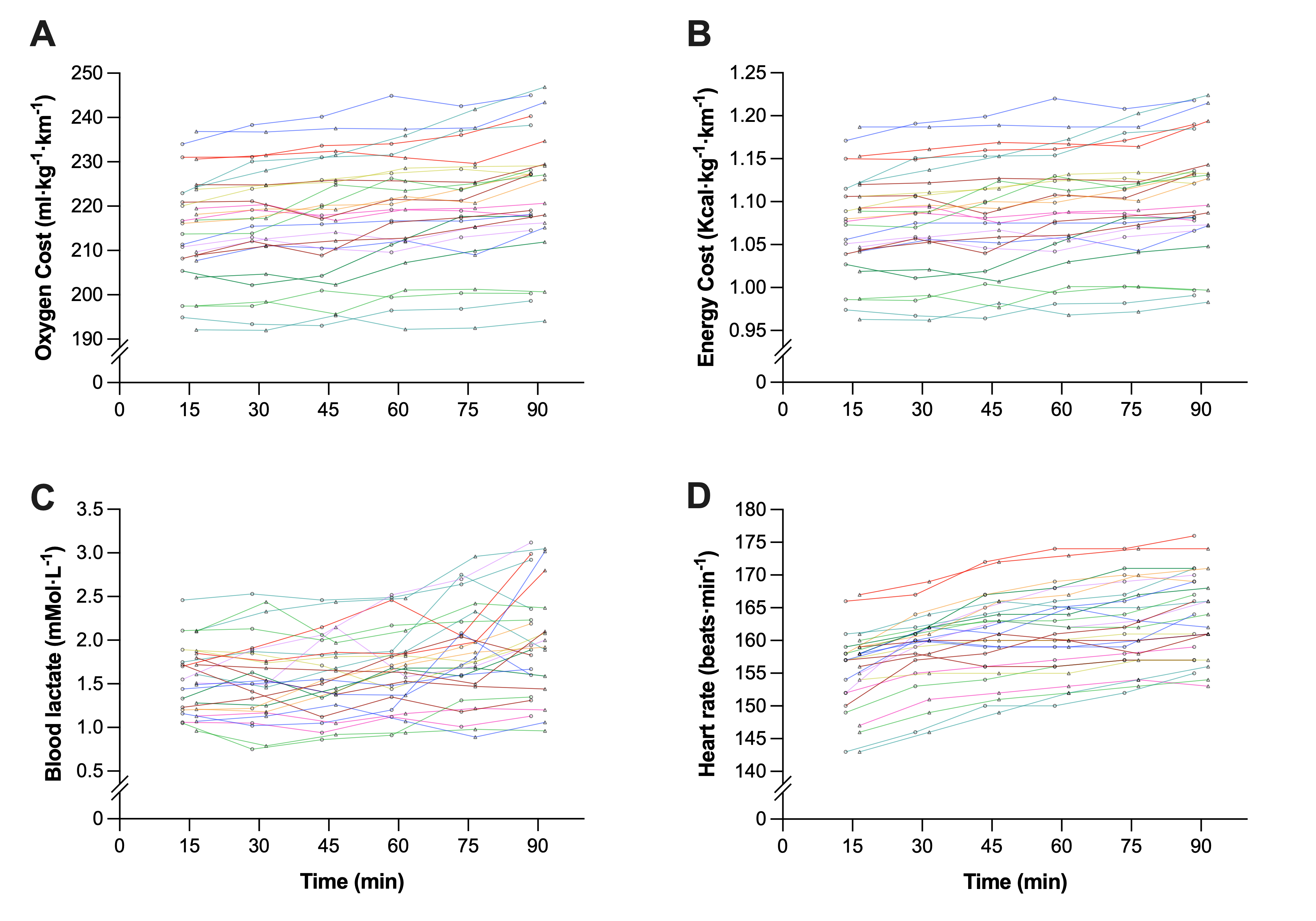
**

**Fig. S1.** Individual responses for oxygen cost (A), energy cost (B), blood lactate (C) and heart rate (D) during the two 90 min runs. Open circles indicate the first run, and open triangles the second. Each participant is represented by different colour (n=14).
